# Emotional responses to suicidal patients and burnout syndrome: the impact of physicians’ mental health on clinical evaluation

**DOI:** 10.1192/j.eurpsy.2025.727

**Published:** 2025-08-26

**Authors:** R. T. Monteiro, P. B. Bolzan, T. P. Comissoli, G. I. Zanella, Y. A. Ferrão

**Affiliations:** 1 Universidade Federal de Ciências da Saúde de Porto Alegre; 2 Serviço de Psiquiatria, Hospital Materno Infantil Presidente Vargas (HMIPV), Porto Alegre, Brazil; 3 Vrije Universiteit Amsterdam, Amsterdam, Netherlands

## Abstract

**Introduction:**

Clinicians’ emotional responses to patients (countertransference) have a significant impact on treatment outcomes. This process is particularly relevant when assessing patients at risk for suicide, as they elicit powerful feelings such as sadness, disapproval, hostility, and distance. These feelings influence risk assessment and have not been the subject of many research studies. Burnout syndrome is a state of complete exhaustion related to work and represents an important factor that impairs the decision-making process. However, there is little existing literature on the impact of physician burnout on emotional responses to suicidal patients. We hypothesized that burnout impacts clinicians’ emotional responses to suicidal patients.

**Objectives:**

This study aims to identify whether burnout influences physicians’ emotional responses (countertransference), comparing psychiatrists and non-psychiatrists.

**Methods:**

An anonymous web-based survey was conducted using the software REDCap and employed a snowball sampling method. Participants were volunteers and could withdraw at any time. The study was approved by the University Ethics Committee. The survey included the Informed Consent Form (ICF), a Sociodemographic Questionnaire, the Burnout Assessment Tool (BAT), and the Rating Scale for Countertransference (RSCT), which evaluates the main emotional responses (approach, indifference, or rejection). Other scales were included but are being developed in separate studies.

**Results:**

Of the 210 respondents, 179 (85.2%) completed the survey: 108 (60.3%) were female, and 166 (92.7%) self-identified as white-colored-skin. The mean age was 37.22 years (SD = 12.33), with a median of 6 years (range: 0 to 48) of professional experience. Among the respondents, 65 (36.3%) were medical residents, and 112 (62.6%) were already specialists; of these, 54 (48.2%) identified as psychiatrists. Physicians with a lower age (r = -0.346), lower income (r = 0.226), and less time since graduation (r = 0.332) experienced more burnout. When evaluating physicians in general, those who experienced burnout tended to exhibit emotional responses of indifference (r = 0.259). When comparing medical specialties, psychiatrists reported less burnout than non-psychiatrists.

**Conclusions:**

We found preliminary evidence that burnout syndrome compromises emotional responses (countertransference), as physicians who experience burnout exhibit more indifference and less approach when evaluating suicidal patients.

**Disclosure of Interest:**

None Declared

